# Windows of opportunity for Ebola virus infection treatment and vaccination

**DOI:** 10.1038/s41598-017-08884-0

**Published:** 2017-08-21

**Authors:** Van Kinh Nguyen, Esteban A. Hernandez-Vargas

**Affiliations:** 1 Frankfurt Institute for Advanced Studies, Ruth-Moufang-Strasse 1, 60438 Frankfurt am Main, Germany; 2 Systems Medicine of Infectious Diseases, Helmholtz Centre for Infection Research, Braunschweig, Germany

## Abstract

Ebola virus (EBOV) infection causes a high death toll, killing a high proportion of EBOV-infected patients within 7 days. Comprehensive data on EBOV infection are fragmented, hampering efforts in developing therapeutics and vaccines against EBOV. Under this circumstance, mathematical models become valuable resources to explore potential controlling strategies. In this paper, we employed experimental data of EBOV-infected nonhuman primates (NHPs) to construct a mathematical framework for determining windows of opportunity for treatment and vaccination. Considering a prophylactic vaccine based on recombinant vesicular stomatitis virus expressing the EBOV glycoprotein (rVSV-EBOV), vaccination could be protective if a subject is vaccinated during a period from one week to four months before infection. For the case of a therapeutic vaccine based on monoclonal antibodies (mAbs), a single dose might resolve the invasive EBOV replication even if it was administrated as late as four days after infection. Our mathematical models can be used as building blocks for evaluating therapeutic and vaccine modalities as well as for evaluating public health intervention strategies in outbreaks. Future laboratory experiments will help to validate and refine the estimates of the windows of opportunity proposed here.

## Introduction

Emerged in 1976, Ebola virus (EBOV) has since caused numerous outbreaks in West African countries infecting ten to hundreds of cases^1^. The recent outbreak in West-Africa (2014–2016) resulted in nearly 30.000 infected cases with one-third of them being fatal^2^. Damages to the vascular systems during infection lead to bleeding, multi-organ failure, hypotensive shock, and death^3^. Ebola virus disease (EVD) displays in a wide range of non-specific symptoms early after infection, making diagnosis and early detection difficult^3^. The infection is acute leading to death within one to two weeks^1, 3^. As a result, complete observations of disease progression or comprehensive evaluations of potential treatment options are problematic.

Experimental observations showed that the immune system often fails to control EBOV infection leading to elevated levels of viral replication^3^. Adaptive immune responses were poor in fatal cases while survivors developed sustained antibody titers^3^. However, follow-up durations were different between fatal cases (approximately one week^1^) and survivors (from a few weeks to months^1^). Currently, treatment of EBOV infection is mainly based on supportive care^4^. Vaccines and therapeutics approaches are still under development and licensure with promising results for certain antivirals^4–6^, passive immunotherapy and vaccination^7–9^.

On one hand, EBOV-infected nonhuman primates (NHPs) treated early with monoclonal antibodies (mAbs) were able to recover after challenged with a lethal dose of EBOV^10^. EBOV-infected patients treated with mAbs in addition to intensive supportive care were also more likely to recover^4^. On the other hand, NHPs vaccinated early with the rVSV-EBOV vaccine survived a lethal EBOV challenge^11^. Based on the rVSV-EBOV vaccine, a recent community trial showed protective efficacy in a ring vaccination design among the group vaccinated early^12^. These results prompted that the outcome of EBOV infection is sensitive to the time of intervention. Failing to catch up with the infection course could alter the chance to survive EBOV infection. Tailoring time windows of intervention is thus critical at both clinical and epidemiological levels.

Building a tractable approach that integrates systematically biological and medical research data is crucial to harness knowledge and to tailor therapeutics and vaccines. In this context, mathematical modeling has been a useful companion approach to advance understandings on mechanisms behind incomplete empirical observations^13^. An overwhelming amount of viral infection modeling studies has been done, e.g., influenza virus^14–18^, human papilloma virus (HPV)^19^, and human immunodeficiency virus (HIV)^20, 21^, among others. These studies provided interpretations and quantitative understandings of the mechanisms that control viral kinetics, which are instrumental to formulate treatment recommendations^20–24^. Although EBOV infection has been endemic in West-Africa countries for decades^1^, modeling studies of EBOV infection are still rare. To the best of our knowledge, the first endeavor to model EBOV replication *in vitro* showed that the within-host EBOV’s basic reproductive number is at least two fold higher than that for influenza virus^25^.

Using *in vivo* experiments in NHPs, this paper aimed to model the interactions between EBOV replication and IgG antibody dynamics, with and without passive immunotherapy. In particular, variations of simple mathematical models representing different interaction mechanisms were fitted to selective parts of the experimental datasets. The goodness of fit of the models was compared when needed to rule out less supportive models. The developed models were then used to estimate the needed time windows to achieve effective interventions, measuring by the ability to inhibit viral replication.

Considering an EBOV infection with a high infective dose, our numerical results showed that a general antibody response dynamic—as if it was stimulated by the rVSV-EBOV vaccine at the day of infection—would rather be late to control EBOV replication. To prevent a potentially lethal infection outcome (i.e., viral load higher than 10^6^ TCID50), a host would need either a high antibody concentration early after infection or a therapeutic in-place sufficiently early to enable the host’s adaptive immune responses to catch up the infection. Simulations of the developed models provided conservative estimates for these critical windows of opportunity. In particular, therapeutic treatments could be effective if an assumed long-lived monoclonal antibody was administrated as late as 4 days post infection. Considering together with a general antibody response dynamic, the rVSV-EBOV vaccine could provide pre-exposure prophylaxis if it was given at least 6 days before exposure. A later vaccination could still be effective, depending on individual infection profile such as infecting with a smaller dose and having a strong antibody response. However, these estimates would be too risky to use in practice. The models also suggested that circulating EBOV-specific antibody could diminish below protection level approximately four months after vaccination. Altogether, the framework presented here could help to tailor appropriate time windows for effective therapeutic and prophylactic interventions when more data are available.

## Results

### Study Design

This study aimed to portray a conservative estimate—as to safety—of the window of opportunity for EBOV treatment and vaccination. By assessing the viral dynamic in cases of infecting with a lethal dose, the possibilities of short-termed stimulation of antibody production^26, 27^, and varied degrees of immune responses strengths, a conservative estimate could be observed. Here, immune responses modeling considered only antibody dynamics due to three main reasons. First, antibody responses have shown to be a consistent and long-lasting protective factor in EBOV infection^3, 11, 12, 28–30^. Second, when innate immune responses might help in controlling EBOV infection, the effect is transient and debatable^3, 31^; furthermore, taking into account this effect or adaptive cell immune responses would only yield a more optimistic estimate. Third, antibody response data have been well-reported in highly controlled experimental studies^11, 29^, facilitating the parameter estimation in modeling processes.

**Figure 1:**
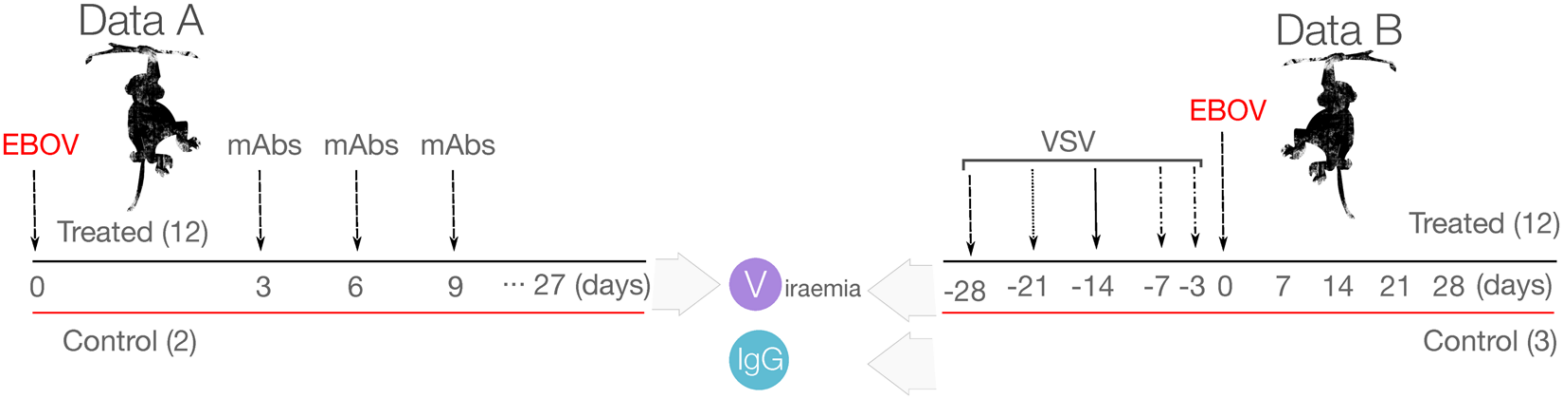
Experimental designs of the two data sources. The experiments were conducted in nonhuman primates (NHPs). The numbers in the parentheses are the sample size. The days zero indicate the time of Ebola virus infection. *mAbs*: time of monoclonal antibody treatments^10^; *VSV*: time of vaccination with the recombinant vesicular stomatitis virus-Zaire EBOV (rVSV-EBOV)^11^.

To construct mathematical models for determining windows of opportunity for both treatment and vaccination, we considered data from two complementary strategies: a passive^10^ and an active immunization protocol^11^ using monoclonal antibodies (mAbs) and the rVSV-EBOV vaccine, respectively. A schematic representation of the NHPs experiments is provided in Fig. 1. Additional experimental details can be found in Materials and Methods. Viral load data in the control and treated cases were extracted from both the studies^10, 11^. The IgG antibody dynamic was available to assess its effects on the viral load^11^ whereas the mAbs dynamic was only available in terms of administrated time points and dosages.

NHPs are considered the best animal model to recapitulate EVD observed in humans^3, 32^. In addition, controlled and defined experimental conditions are key guidances to model the complex interactions between viruses and immune responses, e.g., defined time of infection, consistent sampling time among the subjects, uniform host’s conditions, and consequently immune responses. The viral load was considered to determine the effect of interventions. Epidemiological and pharmacological studies reported that a viral load higher than 10^6^ copies/mL^4, 33^ is associated to a higher mortality rate, whereas observations on experimental data in NHPs showed that animals with viral load levels higher than 10^6^ TCID50 were fatal^10, 11^. Thus, we assumed that subjects with a viral load level higher than this threshold will more likely have a *severe outcome*.

### EBOV replication profile

**Figure 2:**
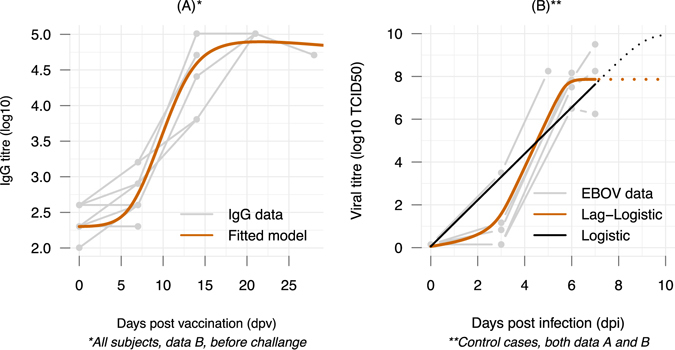
Fitting models to the IgG and viral titer data. Gray lines are subject-specific data. (**A**) Data of the IgG titer post vaccination and prior to EBOV challenge were used to fit to the model of antibody responses. The fitted values of the model were superimposed (orange line) illustrating a general profile of the IgG dynamic after exposure to EBOV. (**B**) Viral titers in control cases form both data sources^10, 11^ were used to evaluate EBOV replication models in treatment-free scenarios. The follow-up data were stopped when the animals reached the endpoints to be euthanized^10, 11^.

EBOV replication dynamics in the absence of interventions were modeled using only EBOV titers (in TCID50) of control cases in the used datasets^10, 11^. We considered two models, including the logistic growth model and a modified logistic growth model as follows

1 $$\begin{array}{ll}{\rm{Logistic}}: & \frac{dV}{dt}={r}_{V}V\,(1-\frac{V}{{K}_{V}})\end{array},$$

2 $$\begin{array}{ll}\text{Lag}{\rm{-}}\text{Logistic}: & \frac{dV}{dt}={r}_{V}V\,(1-\frac{V}{{K}_{V}})\,(\frac{V}{{I}_{n}+V})\end{array},$$

where *r*_*V*_ denotes the virus replication rate and *K*_*V*_ denotes the carrying capacity of the host. The parameter *I*_*n*_ expresses a threshold below which the virus replication is restrained. Both models assume the viral replication is only limited by available resources of the host. Considering a model selection based on Akaike Information Criterion (AIC) (see Materials and Methods), the model Eq. (2) with a lag-phase early after infection (AIC = −10) portrayed the data better than the logistic growth model (AIC = 21) in Eq. (1) (see also Fig. 2B and the parameter estimates in Table S3). EBOV needed approximately three days to gain the momentum to grow exponentially, suggesting this is a crucial period for a successful treatment. Noting that the EBOV replication profile represents the cases infecting with a lethal dose; as such a varied, subject-specific lag-phase as a function of the inoculum could be expected. Nevertheless, in terms of safety, using EBOV dynamics based on a lethal dose to evaluate treatment therapies would provide predictions for worst-case scenarios.

### Antibody profile after EBOV vaccination

To obtain a general IgG dynamic in EBOV infection, IgG data^11^ after EBOV vaccination and before EBOV infection were extracted. This data shows the process from the introduction of EBOV antigen to the secretion of the EBOV-specific IgG antibody without further interventions or infections. Once introduced, the antigen (a live attenuated recombinant vaccine), could be cleared by two main processes: the antigen could be captured by antigen-presenting cells such as macrophages (dendritic cells are reportedly malfunction in EBOV infection^3^) and transported to lymph nodes^34, 35^, or by combining with newly synthesized antibodies forming antigen-antibody (AgAb) complexes—that are then also phagocytosed^34^. This dynamic can be written as follows

3 $$\frac{dAg}{dt}=-{\delta }_{Ag}Ag-{\beta }_{Ag}AgAb$$

where *δ*_*Ag*_ and *β*_*Ag*_ denote the removing rate of the antigen by phagocytic cells and by binding with antibodies, respectively. The processed immunogen will then elicit antibody secretion through a series of events. Briefly, naive B cells are activated by the binding with antigens and experienced the formation of germinal centers in the lymph nodes, leading to B-cell maturation, differentiation, proliferation, and consequently antibody secretion^34, 35^. The time needed for these processes can be summarized as an auxiliary delay state as follows

4 $$\frac{d{G}_{Ag}}{dt}=\frac{{\delta }_{Ag}Ag-{G}_{Ag}}{{\tau }_{Ag}}.$$

Here, we assumed it takes *τ*_*Ag*_ days to process the immunogen to immunogenic signal *G*_*Ag*_. Noting that although antibody production could still be stimulated and remained at a high level for a long time^9^, there are experimental evidences showing that IgG titers decayed quickly after two or six months^26, 27^. To stay on the conservative end, we assumed that antibody production will halt when all the antigens are already cleared. Afterwards, the B-cell proliferation and antibodies secretion can be summarized as

5 $$\frac{dAb}{dt}={r}_{Ab}{G}_{Ag}-{\beta }_{Ab}AgAb-{\delta }_{Ab}Ab$$

where *r*_*Ab*_ reflects the rate of antibody production, and *δ*_*Ab*_ is the natural decaying rate of IgG, which is approximately 28 days^34^. The parameter *β*_*Ab*_ = *ρβ*_*Ag*_ denotes the removing rate of antibodies from binding with the antigens where *ρ* > 0 reflect potential varied stoichiometry in the antigen-antibody interaction^36, 37^. Fitting the model Eqs (3)–(5) to the average IgG data of all subjects resulted in a classic antibody response picture, namely a lag phase follows by an exponential phase before reaching a plateau (Fig. 2A). The EBOV-specific IgG antibody appeared negligible during the first week and a high and steady level can only be acquired two weeks after vaccination.

### Tailoring windows of opportunity for prophylactic vaccines

Experimental observations and modeling studies showed that immune responses dynamics were not dependent on viral dynamics but mainly on the initial stimulation of the immune cells^38–41^. Thus, we assumed the IgG dynamic elicited from a vaccination is independent of the viral replication dynamic. In addition, experimental data showed that challenging vaccinated-NHPs with EBOV briefly boosted the IgG dynamic from the immediate state to a higher level^29^. In the used data^11^, the IgG dynamic also showed a slight boost in some animals after a week. Here, we assumed that EBOV infection would elicit the same IgG dynamic as in the case of vaccination modeled by Eq. (5), i.e., term *Ab* in Eq. (6). Consequently, the IgG dynamic promoted from EBOV infection is accumulated to the IgG dynamic elicited from a vaccine supplied to the host *κ* days prior to the infection, i.e., term *Ab*(*κ* + *t*) in Eq. (6). Numerical simulations showed a good resemblance to the used IgG data (Fig. S1). Considering these altogether, the viral replication model Eq. (2) was extended to

6 $$\frac{dV}{dt}={r}_{V}V\,(1-\frac{V}{{K}_{V}})\,(\frac{V}{{I}_{n}+V})\,(1-\frac{Ab+\omega Ab(t+\kappa )}{{K}_{Ab}}).$$

The parameter *K*_*Ab*_ was introduced to the EBOV replication dynamic (Eq. (2)) to reflect a functional threshold at which the antibody titers inhibit EBOV net growth rate. Crossing this threshold leads to the virus titer being cleared. Only subjects those had been vaccinated *κ* days before the infection have *ω* = 1, otherwise *ω* = 0.

**Figure 3:**
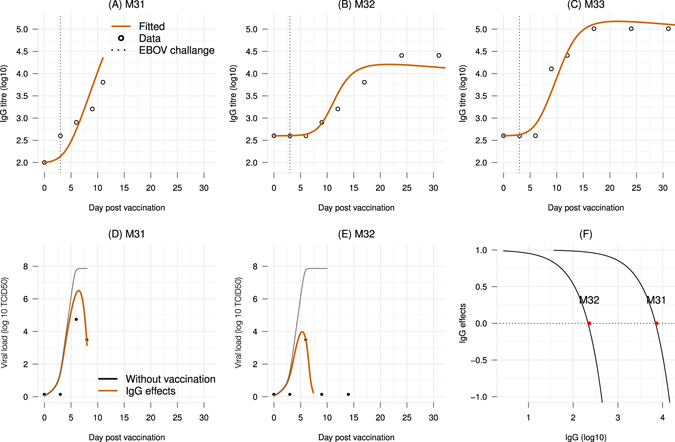
Effects of the IgG antibody on controlling viral load. (**A**–**C**) Fitting the IgG dynamic model Eqs (3)–(5) to the IgG data of the three subjects vaccinated three days before the EBOV challenge. Noting that the M31 had an exceptional low baseline IgG (100) while all other animals had a higher baseline (ranged from 200–400 with a mean of 269). (**D**,**E**) Fitting the viral dynamic model Eq. (6) to the viral load data of the two subjects vaccinated three days before the EBOV challenge. The model without vaccination (solid black line) is added as reference. (**F**) Functions of IgG effect on controlling viral growth in each subject.

To test if this model can reproduce the data, the IgG titer and viral load data of two animals vaccinated three days before EBOV infection were used (M31 and M32); these were the only two animals with detectable viral titers in the experiment^11^. Firstly, the antibody dynamic *Ab* was obtained by fitting the equations Eqs (3)–(5) to the IgG data of the two subjects. The parameter *r*_*Ab*_ was refitted to allow subject-specific responses while fixing the rest of parameters to the previously obtained estimates from the data of all subjects (Fig. 2A, Table S2). Afterwards, the *Ab* outputs were used as inputs to fit the model Eq. (6) to viral titers data of the two subjects. With the assumption that EBOV would replicate similarly among infected subjects, only the parameter *K*_*Ab*_ was estimated while fixing the other parameters to the previously obtained estimates from the model Eq. (2). Figure 3D,E show that the model Eq. (6) could reproduce the viral dynamics in the two subjects (M31 and M32). Different estimates of *K*_*Ab*_ were obtained for each subject, suggesting possibly subject-specific antibody working thresholds.

**Figure 4:**
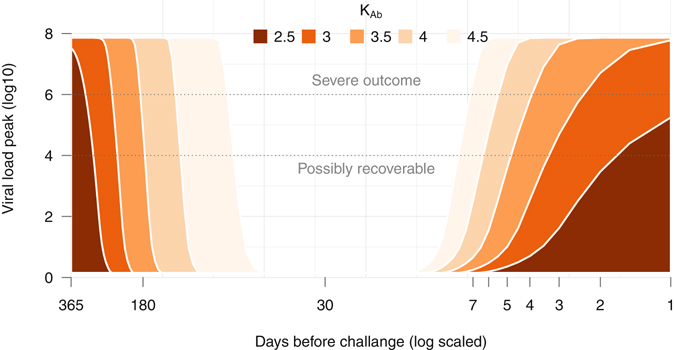
Simulating windows of opportunity for EBOV vaccination. The model Eq. (6) of viral dynamics in the presence of the IgG antibody is used. The x-axis shows the time of vaccination from one year to one day before a subject is challenged with a lethal dose of EBOV. The five shades of color represent the five assumed antibody working threshold (*K*_*Ab*_)—the darker the stronger the antibody’s effect in inhibiting the viral replication. Each combination of the vaccination time and the working threshold was used to generate the viral replication dynamic from which its peak was retrieved and plotted in the y-axis. For example, the white area indicates the period when the vaccination completely represses the viral replication with *K*_*Ab*_ = 4.5. The *severe outcome* indicates the viral load level that was associated with lethal outcome in EBOV infection in both human^33^ and NHPs^4, 10, 11^. It was shown that a viral load reaching 10^4^ TCID50 can still be recoverable but not entirely^10^.

By varying the time of vaccination *κ*, a time window during which vaccination could prevent a likely-lethal viral load level can be estimated. Since the working threshold of the antibody response (*K*_*Ab*_) can be subject-specific (Fig. 3F), a range of thresholds based on the observed IgG data from 10^2.5^ to 10^4.5^ were tested. Based on data of the control cases (Fig. 2B) and empirical observations in EBOV-infected human^33^, a subject with a viral load level higher than 10^6^ could be considered as having a severe outcome. Figure 4 shows the time windows for different antibody working threshold *K*_*Ab*_ and the vaccination time *κ*.

For each *K*_*Ab*_, there is a safe time window where viral titers cannot be observed. The higher the IgG concentration required to suppress viral replication (*K*_*Ab*_), the narrower the time windows for a successful intervention. For example, a threshold *K*_*Ab*_ = 10^4^ could prevent EBOV replication from reaching severe viral load levels if only a subject had been vaccinated at least 6 days before infection. However, if the subject had received vaccination for more than four months before, the circulating antibody level could have decreased below the required working threshold (*K*_*Ab*_) at the time of infection. As such, if secondary antibody responses to EBOV infection are not considerably faster than primary responses, the subject would also succumb to the disease. Here, the secondary antibody response to EBOV infection was assumed similar to the primary response model in vaccination and that the IgG titer was accumulated to the primary response (Fig. S1).

Remarkably, assuming an infected subject would develop the same IgG profile as a vaccinated subject at the day of challenge, simulation results showed that the general IgG response would fail to keep the viral load from reaching its peak, regardless of the working threshold *K*_*Ab*_ (Fig. S2). At best, the general IgG profile developing from the day of infection could only clear the virus 9 days post infection, if the subject is still alive after several days withstanding massive viral titers.

### Tailoring window of opportunity for therapeutic vaccines (mAbs)

In the experiment with passive antibody treatment^10^, viral dynamics in the animals treated with mAbs ceased after the first dose on day 3 post infection. Although this may include the role of host’s immune responses, the previous section has shown that the general IgG profile starting at the day of infection may not able to clear the virus at least until day 9 post infection (Fig. S2) and that the antibody level was negligible during the first few days. Subsequently, two further doses of mAbs were given on day 6 and 9 post infection, effectively clearing the viral replication in all but one animal^10^. Thus, for EBOV-infected subjects, the mAbs treatment would play a decisive role in tackling EBOV infection during the first days after infection. To recapitulate the viral dynamics in subjects treated with mAbs when antibody responses were negligible, the viral dynamics are rewritten as follows

7 $$\frac{dV}{dt}={r}_{V}V\,(1-\frac{V}{{K}_{V}})\,(\frac{V}{{I}_{n}+V})\,(1-{K}_{m}\frac{M}{1+M})$$

8 $$\frac{dM}{dt}=-{\lambda }_{M}M,\quad M\mathrm{(0)}=0$$

where *M* is the concentration of mAbs that was administrated impulsively with a fixed dose (see Materials and Methods). The mAbs dynamics are denoted by $M\in {\mathbb{R}}$, a set of instants *T* = {*τ*_*k*_}, ${\tau }_{k}\in {\mathbb{R}}$, *τ*_*k*_ < *τ*_*k*+1_, *k* = 1, 2, …. At each *τ*_*k*_, *M* is changed impulsively by *M*(*τ*+) = *M*(*τ*−) + *M*_0_(*τ*). In other words, impulsive system dynamics are governed by its ODE Eq. (8) when *t* ≠ *τ*_*k*_. Only at the instant *τ*_*k*_, *k* = 1, 2, …, the state variable is instantaneously changed from *M*(*τ*−) to *M*(*τ*+) = *M*(*τ*−) + *w*. In the experiment^10^, a fixed mAbs dose (*w* = 50) was administrated at three time points ${\tau }_{k}=[3,6,9]$. Here the mAbs are assumed acting indifferently from the IgG antibody, i.e., inhibiting the viral replication. Noting that since the mAbs data were not recorded but only the administrated dose was available^10^, the mAbs concentration was normalized in Eq. (7) to have a neutral unit. The combination of parameter *K*_*m*_ and the dynamics of the mAbs leads to a similar working threshold mechanism as in Eq. (6).

Evaluations of the model Eqs (7) and (8) were done by fitting to the viral dynamics data, separately for each treated animal with an observable viral load^10^. As the model does not consider the effects of antibody responses, only the viral load data from day 0 to day 9 were used. Note that two animals, A3 and B3, were excluded from modeling studies: the subject A3 had no records of viral load after challenged with EBOV before any treatments were given—providing no information on the treatment effect; and the subject B3 was the only non-survivor for reasons currently unknown despite treatments^10^. The parameters *r*_*V*_, *K*_*V*_, and *I*_*n*_ were fixed to the earlier estimates in Eq. (2). For simplicity, the mAbs are assumed to have a stable and long elimination half-life of 28 days across the subjects^42^.

**Figure 5:**
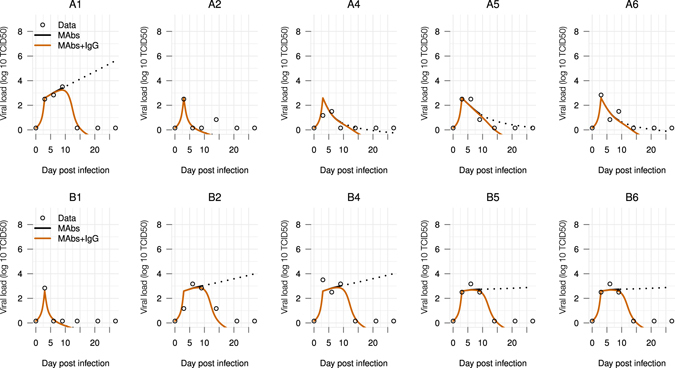
Fitting the mAbs treatment effect model. *mAbs*: fitted model with only mAbs effect during the first nine dpi, dashed lines show the extrapolated viral load kinetics from this model; *mAbs*-*IgG*: adding the general IgG profile with the working threshold *K*_*Ab*_ = 10^4.5^. mAbs half-life is 28 days. Two different combinations of monoclonal antibodies were tested in NHPs (ZMapp1 and ZMapp2)^10^. The top panel of figures (**A1**–**A6**) presents the six NHPs receiving three doses of ZMapp1, while the bottom panel of figures (**B1**–**B6**) presents the six NHPs receiving three doses of ZMapp2^10^.

Figure 5 shows that the model portrays adequately the viral load kinetics in all subjects. Interestingly, the mAbs treatment effect seemed to be separated in two groups: a low effect group (*K*_*m*_ < 1) that allowed viral titers to linger until day 9 and a high effect group (*K*_*m*_ > 1) that quickly stemmed down the viral titers (details in Table S3). Extrapolating the model Eq. (7) to time points after day 9 post infection showed a sustained viral load in those subjects whose the mAbs effect is low (*K*_*m*_ < 1), see Fig. 5. Because the mAbs were already assumed having the longest elimination half-life observed in natural antibodies, this result suggests that mAbs treatment alone may be insufficient for those that the mAbs effect is low. To take into account the effect of the host’s IgG response to EBOV infection, we incorporated the general IgG profile developed earlier into the model Eq. (7) and simulated the viral load dynamics with a conservative working threshold 10^4.5^ in each subject as follows

9 $$\frac{dV}{dt}={r}_{V}V\,(1-\frac{V}{{K}_{V}})\,(\frac{V}{{I}_{n}+V})\,(1-{K}_{m}\frac{M}{1+M}-\frac{Ab}{{K}_{Ab}}).$$

Here, it was continued to assume that EBOV-infected subjects would able to develop a similar IgG profile as in those subjects vaccinated with the rVSV-EBOV vaccine^11^. Figure 5 shows that including the effects of IgG into the treatment model replicated closely the viral load data, suggesting that the host’s antibody response would have played a key role in resolving the EBOV infection for those subjects that mAbs treatment was not sufficient.

**Figure 6:**
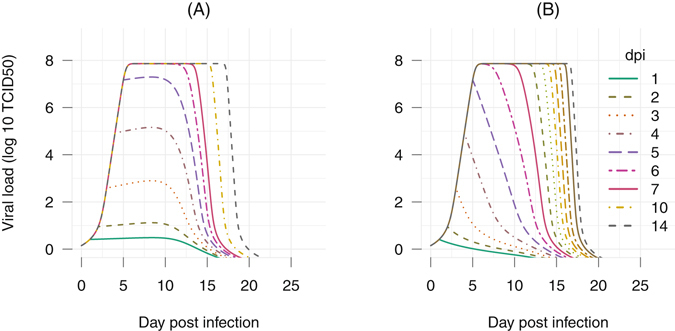
Simulation of single-dose mAbs treatment assuming a long mAbs half-life of 28 days. The time administrated mAbs were varied from 1 to 14 days. For each regimen, the model of viral dynamic including both treatment and IgG effects were simulated to generate the corresponding viral load dynamics. (**A**) assuming a low effect (*K*_*m*_ = 0.98), the general IgG profile with the working threshold *K*_*Ab*_ = 10^4.5^, and a long half-life mAbs of 28 days; (**B**) assuming a high effect (*K*_*m*_ = 1.47), the general IgG profile with the working threshold *K*_*Ab*_ = 10^4.5^.

In light of these results, therapeutic treatment windows can also be developed using the model Eqs (8) and (9). For illustration purpose, we varied the time of treatment administration to define which treatment initiation can prevent the viral load to reach fatal levels, i.e., viral load higher than 10^6^ TCID50. Figure 6 illustrates EBOV kinetics considering a single dose treatment approach, we can observe that a single dose of a long-lived mAbs administrated up to day 4 post infection could clear the virus titers before it reached the likely-lethal level.

## Discussion

The recent unprecedented EBOV outbreak in West Africa has affected more people than in all previous outbreaks combined. While significant progresses have been made in therapeutics and vaccines against EBOV on a preclinical level, no licensed products are currently available. Lack of market for products, consequently interests of pharmaceuticals companies, could have hindered the progress. Furthermore, the high pathogenicity of EBOV hampers the possibilities to have comprehensive data and to conduct clinical and efficacy studies in EBOV infection. In this context, using mathematical models in combination with experimental data can be essential for EBOV countermeasure developments.

This paper modeled EBOV replication dynamic in situations with and without interventions using NHPs data. These sources of data possess advantages that are unaffordable in human settings: the ability to conduct a desired experiment with controlled and known conditions. For example, infective dose, time points of infection and of measurements after infection can be recorded in high precision. However, the results need to be interpreted with caution as unexpected differences when applying the results to human settings are inevitable. In general, however, immune responses from NHPs are in close similarity to human and NHPs have been a gold standard model for understanding human infections^3, 32, 43, 44^. This was continued to show working with the recent successful in applying the rVSV-EBOV vaccine in human^9, 12^. It is important to point out that in this paper all vaccinated and infected subjects were assumed able to mount the general antibody response. Therefore, the results are not applicable for potentially immunocompromised subjects.

Because antibody responses showed to be a consistent protective factor in EBOV infection^3, 11, 12, 28–30^, antibody dynamics were considered in mathematical modeling. Antibody response data were also the only well-reported part of the immune systems in highly controlled experimental studies^11, 29^. Nevertheless, other parts of the immune systems could still play a role in EBOV infection. There are suggestions of potential effects of innate immune responses on early control of EBOV infection^3, 31^, which our models do not take into account. Based on our primary assumption to provide worst case scenarios to EBOV infection, taking into account the effect of innate immune responses or adaptive cell immune responses would only result in more optimistic scenarios to vaccination. In addition, experimental data^10, 11, 31^ showed that in cases of no interventions, EBOV titers exhibits the same dynamic as our model, i.e., a very low viral titer during the first three days and a quick increase to 10^6^–10^7^ TCID50 on day 6 post infection. Simulations of our model with IgG effects (Fig. S2) also reproduced experimental viral dynamics in which the viral load could be cleared from day 9 onward^31^.

Modeling the IgG dynamic showed that it took approximately three days to elicit EBOV-specific antibody (Table S1). This agrees with observations in literature^35^ in which antibody-secreting cells appear on 3–4 days after infection. Our results suggested that even if a host effectively produces the general antibody profile (Fig. 2A) after infection, the pace of the response would still be too slow to counteract EBOV replication, which needs three days to start an exponential growth (Fig. 2B). As the IgG antibody titer appeared negligible during the first week post vaccination (Fig. 2A), during this period the innate immune responses are also largely ineffective^3^, the protection to EBOV infection appeared depending on having a high level of antibodies—as all animals vaccinated sufficiently early were survived^11^. Differences in the race between the EBOV replication and the antibody response highlight the importance of timeliness in EBOV vaccination: the sooner the better, but might be not too soon. Simulation results showed that the window of opportunity for an effective intervention can be limited, ranging from a few days to four months or more, depending on the immune responses strength and on the infective dose. To be on the safe side, a subject infected with a high dose and has the general IgG response would require to be vaccinated from a week up to four months before the exposure.

The left side estimate of the window (Fig. 4) appeared to exist when vaccinated-NHPs re-challenged at day 113 (≈four months) were all survive while vaccinated-NHPs re-challenged at day 234 (≈eight months) were all death^29^. In human, there exists inconsistent results on the longevity of the antibody when some studies showed sharp drop-offs in antibody tite﻿rs ﻿after two^27^ or six^26^ months while others showed antibody titers remained unchanged up to 360 days^9^. This prolonged existence of the EBOV-specific antibody can promisingly extend the window’s left side, postulating more optimistic opportunities for EBOV prevention. Nonetheless, to ensure a high protection effect, we aimed to the conservative end by assuming that antibody production will stop once the antigens are cleared. Ideally, when longer follow up data with the observation of decaying in EBOV-specific IgG become available, remodeling of the IgG dynamic would fine-tune the window’s left side estimate. The right side estimate of the window (Fig. 4) reflects experimental observations^11, 29^ in which the less risky time of vaccination to achieve pre-exposure prophylaxis is at least one week before exposure. As this estimate was based on a general infection profile infecting with a lethal dose, individual variations could make a late vaccination appear partially protecting. In these cases, however, the protection effect cannot be ascertained for everyone. Attempts to use rVSV-EBOV as a post-exposure treatment are thus risky as also discussed elsewhere^31^.

Combination of mAbs represents one of the most promising therapeutic modality^4^ for EBOV infection. Our results showed that an early use of this supportive treatment is crucial in preventing a fatal outcome in unvaccinated subjects. However, subject-specific responses to the mAbs can be expected. When the role of a host’s antibody response was neglected, mAbs treatment could clear the virus in some but not all the subjects (Fig. 5). Both, long and short half-lives, exhibited the possibility of a viral rebound if the antibody response was neglected (Figs S3 and 5). These results reiterate the key role of the host’s antibody response in clearing the virus once the treatment effect wears off. As the elimination half-life estimate of the used mAbs was not reported, it was not possible to narrow down the estimates of the treatment time window. For example, a half-life of half an hour can also produce the viral load data (Fig. S3) but the estimated drug effect was rescaled (Table S3). Thus, this result needs to be refined when common pharmacokinetics parameters become available, such as the elimination half-life for the mAbs. Here, the model submits a framework to evaluate EBOV treatment regimens in the future.

In summary, this paper proposed mathematical models by using selective parts of different data sources for model evaluation, resulting in a general framework for the development of treatment regimens and vaccination strategies. On top of that, public health policies and initiatives can also be evaluated with realistic treatment efficacy and subject-specific immune responses. In the scarcity of data, mathematical modeling approaches posit a strong potential to uncover useful information in controlling infectious diseases.

## Materials and Methods

### Experimental data

Experimental data considering a therapeutic vaccine using monoclonal antibodies (mAbs) was taken from ref. 10. The mAbs were engineered to specifically recognize the EBOV glycoprotein (GP) inserted in the membrane of the viral particle. In this experiment, a group of 12 macaques was administrated mAbs intravenously at day 3, 6, 9 post infection with a constant dose of 50 mg/kg. These were divided into two groups, 6 NHPs received the mAbs combination ZMapp1 (Group A) and the other 6 received ZMapp2 (Group B)^10^. No treatment was given to the two control cases. EBOV titer increased rapidly but ceased when the first dose of mAbs was administrated. The viral load continued to increase in the control cases until the subjects were euthanized at day 7 post infection. All animals cleared the virus from day 10 onward, with the exception of A1 which presented a high clinical score.

Experimental data for a prophylactic vaccine based on the recombinant vesicular stomatitis virus expressing the EBOV GP (rVSV-EBOV) was taken from ref. 11. In this experiment, groups of two or three macaques were vaccinated at 3, 7, 14, 21, and 28 days before EBOV challenge. Macaques were immunized with a single intramuscular injection of 5 × 10^7^ plaque-forming units (PFU) of rVSV-EBOV. An ineffective vaccine (the VSV-Marburg virus vaccine (VSV-MARV)) was given to three control cases. IgG titers were measured regularly four weeks before and after the challenge. All the vaccinated animals showed a sharp increase of IgG titers one week after vaccination. IgG titers sustained at the level above up to two months. EBOV titers were monitored up to 9 days after the challenge. All the control cases showed a high level of viral titers and were euthanized five to seven days after infection. Viral titer was not observed in all the animals vaccinated at least seven days before the challenge. Among three animals vaccinated three days before the challenge, two had observable viral load in which one died and the other survived. A schematic representation of both NHPs experiments is provided in Fig. 1.

### Model fitting and selection

Selective parts of the datasets were used to evaluate models representing different mechanisms. When applicable, a model comparison was done using Akaike information criteria (AIC). When data are available, extra components involved in the models were computed as forcing functions using a linear approximation. These functions were used as inputs in model fitting instead of adding extra model equations. Time points when there were no measurable viral load were imputed as 10^0.15^ TCID50^45^. Model fitting was conducted in log ten for both the states and parameters. The objective function was defined as the root mean square error of the fitted value and the experimental data. Optimization was done with the Differential Evolution algorithm using the recommended configurations^46^. All simulations were done using R^47^. Details of model fitting can be found in Table S1.

## Electronic supplementary material

Supplementary File
