# Supplementary material for: Windows of opportunity for Ebola virus infection treatment and vaccination
Source: Sci Rep. 2017 Aug 21;7:8975. doi: 10.1038/s41598-017-08884-0 (PMC5567060; doi:10.1038/s41598-017-08884-0)
Supplement: Supplementary file 1 — Supplementary File [file 41598_2017_8884_MOESM1_ESM.pdf]

Supplemental Materials:
Windows of opportunity for Ebola virus infection treatment and vaccination

Van Kinh Nguyen[1,2] **and Esteban A. Hernandez-Vargas**[1,2,*]

[1]Frankfurt Institute for Advanced Studies, Ruth-Moufang-Strasse 1, 60438, Frankfurt am Main, Germany
[2]Systems Medicine of Infectious Diseases, Helmholtz Centre for Infection Research, Braunschweig, Germany
[*]vargas@fias.uni-frankfurt.de

ABSTRACT

In this paper, we employed experimental data of EBOV-infected nonhuman primates (NHPs) to construct a mathematical framework for determining windows of opportunity for treatment and vaccination.

Table S1. Details of the model fitting process and the corresponding results.

Figure	Eqs.	Initial condition	Estimated	Fixed	Note	#
2A	1	5×10^7, see[1]	δ_{Ag}, β_{Ag}			(a)
	2	0	τ_{Ag}			(b)
	3	0	r_{Ab},β_{Ab}	δ_{Ab}, see[2]		(c)
2B	4	$10^{0.15}$, see[3]	r_V, K_V			(d)
	5	$10^{0.15}$	r_V, K_V, I_n			(e)
3A-C	1,2,3	as in (a)-(c)	r_{Ab}	δ_{Ab}, δ_{Ag}, β_{Ag}, β_{Ab} , τ_{Ag} to (a)-(c)		(f)
3D-E	6	as in (e)	K_{Ab}	r_V, K_V, I_n to (e)		(g)
4	6	$10^{0.15}$	None	Ab*		(h)
5:mAbs	7	$10^{0.15}$	K_M	r_V, K_V, I_n		(i)
	8	0	λ_M			(j)
5:mAbs+IgG	9	$10^{0.15}$	None	r_V, K_V, I_n to (e), Ab*, K_M to (i), λ_M to (j)		(k)
6	9	$10^{0.15}$	None	r_V, K_V, I_n to (e), Ab*, K_M to (i), δ_M to (j)		(l)
7	6	Varied	None	Ab* to (c)	Varied K_{Ab}	(m)
S1	6	$10^{0.15}$	None	Ab* to (c)	Varied K_{Ab}	(n)
S2	7,8,9	as in (i)-(k)	-	-	-	(o)

*outputs from the model equations (a)-(c) and the assumption as in Fig. S1

Table S2. Parameter estimates of the model Eqs. (3) to (5) fitted to three subjects vaccinated three days prior EBOV challenge. δ_{Ab} is fixed from literature at 0.0248[2]. The parameters δ_{Ag}, β_{Ag}, and β_{Ab} were fixed from the estimates from the general IgG profile of all subjects (Fig. 2A). Consequently, the parameter r_{Ab} was refitted to allow subject-specific responses.

	δ_{Ag}	β_{Ag}	β_{Ab}	τ_{Ag}	r_{Ab}
All data (Fig. 2A)	1.1187	0.0000	0.0263	3.1574	0.0815
M31	–	–	–	–	0.2195
M32	–	–	–	–	0.0163
M33	–	–	–	–	0.1547

Table S3. Fitting the mAbs treatment effect model. K_{m_1} estimates assumed mAbs half-life (λ_M) is 28 days; K_{m_2} estimates assumed mAbs half-life (λ_M) is half an hour.

Subject	r_V	K_V	I_n	K_{m_1}	K_{m_2}
A1	5.4107	72880400	15.0494	0.9560	1.1646
A2	–	–	–	1.8551	2.2533
A4	–	–	–	1.2655	1.5402
A5	–	–	–	1.1291	1.3752
A6	–	–	–	1.2253	1.4872
B1	–	–	–	1.8551	2.2533
B2	–	–	–	0.9853	1.2003
B4	–	–	–	0.9850	1.2
B5	–	–	–	1.0049	1.2242
B6	–	–	–	1.0049	1.2242

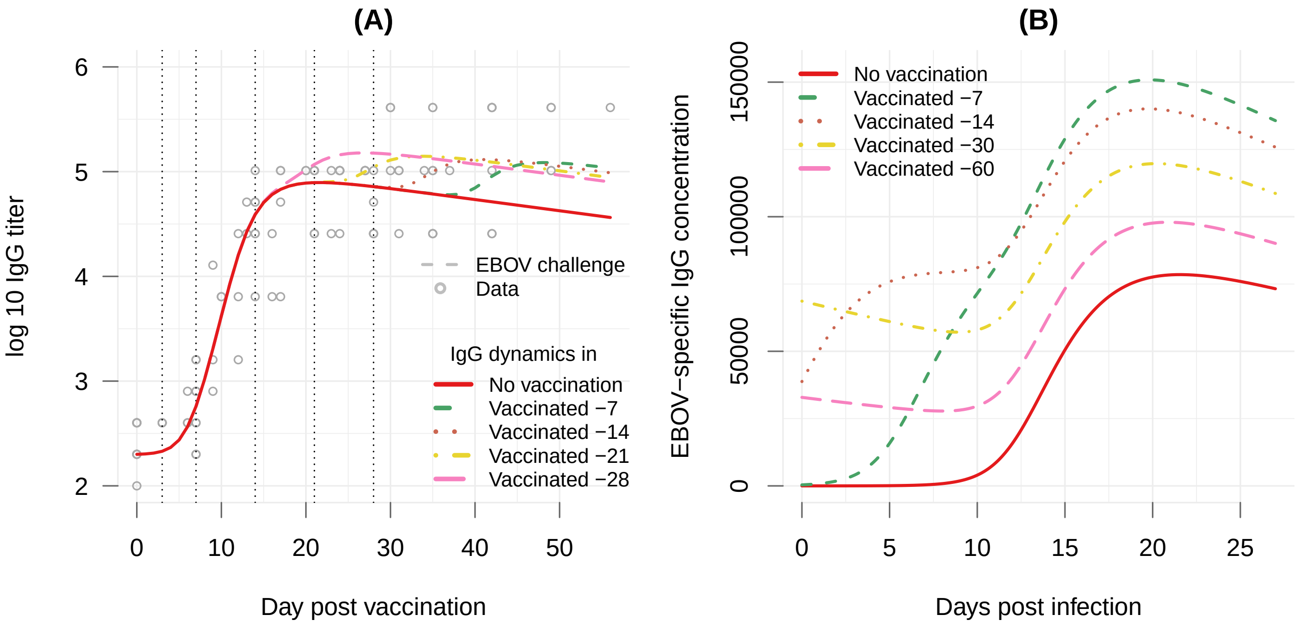

Figure S1. Simulations of the IgG dynamic in different vaccination time and the effect of infection in boosting the IgG dynamics. Minus sign indicates vaccination time (in days) before the day of infection (day zero). **A**: Plotting simulated IgG dynamics against experimental data[1]. **B** Plotting simulated EBOV-specific IgG.

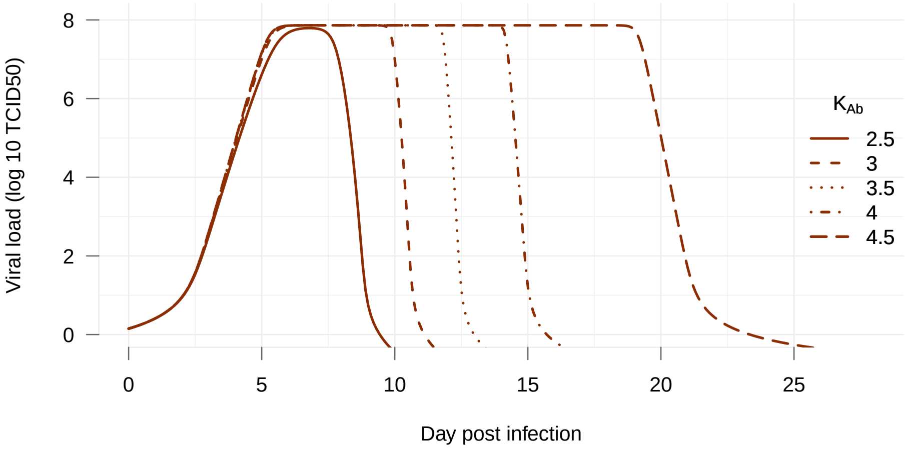

Figure S2. Simulation the general IgG response profile versus viral dynamics. Assuming a normal viral replication rate and an average IgG response profile. The model of viral dynamic including the effect of IgG were simulated to generate the corresponding viral load dynamics.

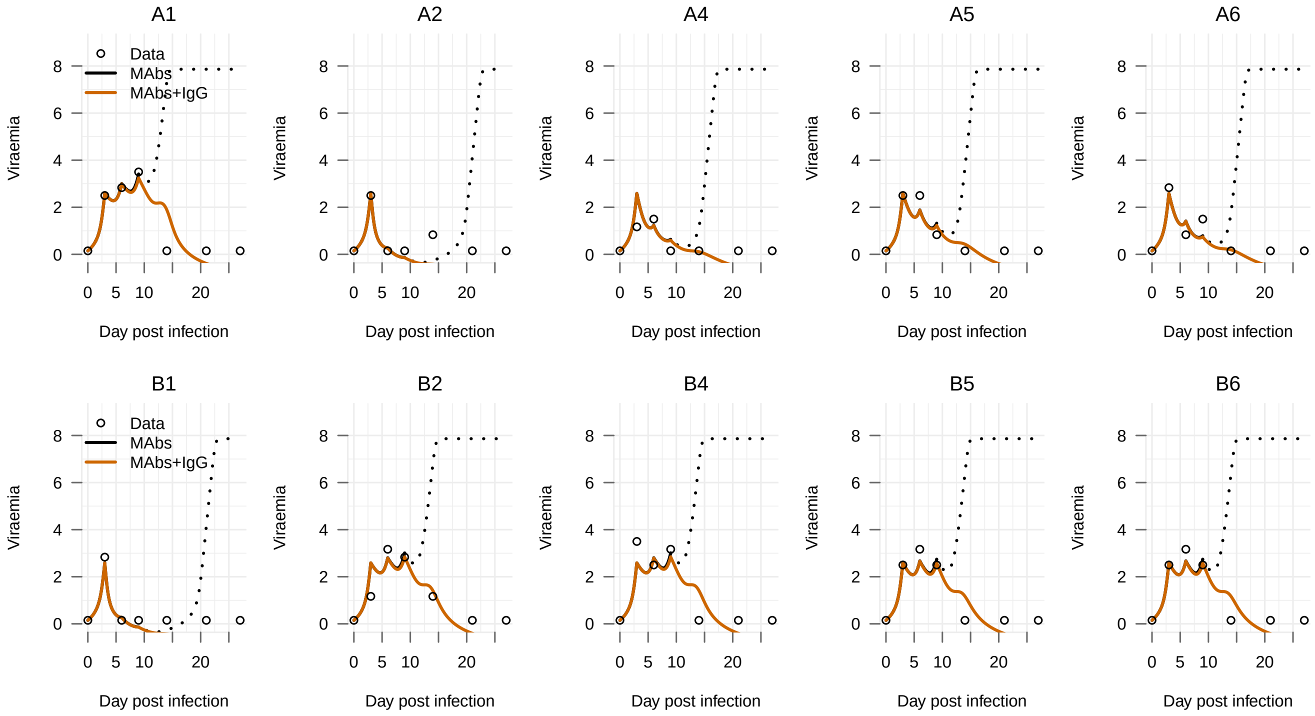

Figure S3. Fitting the mAbs treatment effect model. *mAbs:* fitted model with only mAbs effect during the first nine dpi, dashed line shows the extrapolated viral load kinetics from this model; *mAbs-IgG:* adding the general IgG profile with the working threshold $K_{Ab} = 10^{4.5}$. The assumed mAbs half-life is half an hour.

References

1. Marzi, A. *et al.* VSV-EBOV rapidly protects macaques against infection with the 2014/15 Ebola virus outbreak strain. *Sci.* **349**, 739–742 (2015). DOI 10.1126/science.aab3920.

2. Abbas, A. K., Lichtman, A. H. H. & Pillai, S. *Cellular and Molecular Immunology* (Elsevier Health Sciences, 2011).

3. Chan, K. H. *et al.* Analytical Sensitivity of Seven Point-of-Care Influenza Virus Detection Tests and Two Molecular Tests for Detection of Avian Origin H7N9 and Swine Origin H3N2 Variant Influenza A Viruses. *J. Clin. Microbiol.* **51**, 3160–3161 (2013). DOI 10.1128/JCM.01222-13.
